# How to Prevent and Mitigate Hypersensitivity Reactions to Biologicals Induced by Anti-Drug Antibodies?

**DOI:** 10.3389/fimmu.2021.765747

**Published:** 2021-11-01

**Authors:** Alessandra Vultaggio, Margherita Perlato, Francesca Nencini, Emanuele Vivarelli, Enrico Maggi, Andrea Matucci

**Affiliations:** ^1^ Immunoallergology Unit, Careggi University Hospital, Florence, Italy; ^2^ Department of Clinical and Experimental Medicine, University of Florence, Florence, Italy; ^3^ Translational Immunology Unit, Immunology Area, Pediatric Hospital Bambino Gesù, Istituto di Ricovero e Cura a Carattere Scientifico (IRCSS), Rome, Italy

**Keywords:** anaphylaxis, immunogenicity, hypersensitivity reactions, drug desensitization, anti-drug antibodies, IgE

## Abstract

Biologicals are widely used therapeutic agents for rheumatologic diseases, cancers, and other chronic inflammatory diseases. They are characterized by complex structures and content of variable amounts of foreign regions, which may lead to anti-drug antibodies (ADA) development. ADA onset may limit the clinical usage of biologicals because they may decrease their safety. In fact they are mainly associated with immediate hypersensitivity reactions (HSRs). Development of ADAs is reduced by concomitant immunosuppressive treatment, while it is increased by longer intervals between drug administrations; thus, regular infusion regimens should be preferred to reduce HSRs. Once ADAs have formed, some procedures can be implemented to reduce the risk of HSRs. ADAs may belong to different isotype; the detection of IgE ADA is advisable to be assessed when high and early ADAs are detected, in order to reduce the risk of severe HRs. In patients who need to reintroduce the biological culprit, as alternative therapies are not available, drug desensitization (DD) may be applied. Desensitization should be conceptually dedicated to patients with an IgE-mediated HSR; however, it can be performed also in patients who had developed non-IgE-mediated HSRs. Although the underlying mechanisms behind successful DD has not been fully clarified, the DD procedure is associated with the inhibition of mast cell degranulation and cytokine production. Additionally, some data are emerging about the inhibition of drug-specific immune responses during DD.

## Introduction

Biologicals are indispensable therapeutic agents in immunological, oncological, and inflammatory diseases, and their application in clinical practice is increasing. However, despite the therapeutic benefits, biologicals may cause hypersensitivity reactions (HSRs) that represent a major safety concern and a significant challenge for clinicians. Hypersensitivity reactions may occur during the first lifetime exposure or after repeated administrations and different pathogenic mechanisms are involved. In addition, immediate and delayed HSRs have been conveniently described; immediate reactions occur during the drug administration or within 1 h after the end of infusion, whereas delayed reactions appear from 1 h to several days after (1, 2).

The potential risk of biologicals’ immunogenicity is an important issue in clinical practice, leading to the development of anti-drug antibodies (ADA). Immunogenicity is expected following treatment with non-human sequence proteins, but it is now well established that the immune response may be elicited also by fully human sequences. The unexpected and unpredictable unwanted immunogenicity may impact both the efficacy and the safety of the drug. The induced ADA may compromise the clinical efficacy by altering the circulating drug levels and/or neutralizing its biological functions; furthermore, ADA development has been associated with the onset of HSR, ranging from mild to severe grade. Patients with ADA, as IgG or IgE developed during treatment with biologicals or preexisting, are more likely to have increased risk of immediate HSRs (1).

In patients with HSR toward a biological, approaches for avoiding future adverse events differ depending on the mechanism of reactions. These strategies are very important when no alternative therapies are available. Similarly, it was shown that in some cases, reactions against the biological could be predicted by the presence of ADA.

In this review we discuss the clinical and diagnostic strategies to prevent and mitigate HSR toward biologicals, especially when related to the development of immunogenicity.

## Mechanisms of Hypersensitivity Reactions to Biologicals

Taking into account the structural characteristics of biologicals, which differ from traditional drugs, and their ability to elicit ADA, the mechanisms underlying HSR to biologicals can be divided into ADA and non-ADA mediated. Additionally, ADA may belong to different isotypes, so ADA-mediated HSR may be classified as IgE- and non-IgE-mediated reactions. According to clinical manifestations, immediate HSRs are classified as mild, moderate, or severe, and in some cases even life-threatening reactions have been described (3). Hypersensitivity reactions may have similar clinical presentation in ADA-positive and ADA-negative patients.

### IgE-Mediated Reactions to Biologicals

The development of IgE ADA has been described in patients treated with different types of biologicals (4–9). IgE binds to mast cells’ and basophils’ surface receptors (FcεRI, high-affinity receptor), thus initiating immediate HSRs sustained by the release of vasoactive mediators such as histamine, tryptase, leukotrienes, prostaglandins. IgE ADA are detectable in a subgroup of patients with HSR to biologicals; in patients suffering from immunomediated inflammatory diseases and treated with infliximab who had experienced a previous reaction, about 20% of them tested positive for IgE ADA (10). The onset of an anti-drug humoral response of IgE isotype is facilitated by the repeated administrations of biologicals, so IgE-mediated reactions occur during or after the second drug administration. Cetuximab, largely used in head and neck cancers, represents an exception in this field, because most of the cetuximab-induced reactions occur within minutes upon first treatment exposure (11). Cetuximab-specific IgE antibodies pre-exist to the first drug administration, and their production has been shown to be triggered by tick bites (12). IgE response to cetuximab is different from typical IgE responses specific for protein epitopes expressed by other biologicals; in fact, it is directed towards a mammalian oligosaccharide epitope, galactose-alpha-1,3-galactose (alpha-gal), present in the Fab portion of this monoclonal antibody, as well as present on non-primate mammalian proteins (13). IgE sensitization towards biologicals is associated with a higher severity of reactions, as shown for both infliximab and cetuximab (10, 14), and at least for infliximab, it has been shown more frequently at re-exposure after a period of drug interruption (10). Finally, IgE ADA are more frequently developed in patients with higher ADA levels and earlier ADA onset, but their rate of negativization is faster (15).

### Non IgE-Mediated Reactions to Biologicals

The IgE antibody pathway had been universally accepted as the only pathogenic explanation of anaphylaxis until more recent observations obtained in mouse models suggest the existence of a non-classical (non-IgE-mediated) pathway for anaphylaxis (16). Taking into account that the majority of ADA belong to the IgG isotype, and that the majority of patients tested negative for IgE ADA, the existence of IgG-mediated HSR to biologicals has been hypothesized. Animal models revealed that anaphylaxis may occur through an IgE-independent manner, involving specific IgG, FcγRIII, macrophages, basophils, and the Platelet Activating Factor (PAF) as major mediator (17). The development of drug-specific IgG may lead to the formation of immunocomplexes between biological and ADA with subsequent Complement activation and production of anaphylatoxins (C3a and C5a), which directly activate mast cells, expressing C3a and C5a receptors. Additionally, FcγR-mediated activation of basophils, neutrophils, monocyte/macrophages induced by ADA may be involved in systemic immediate HSR (18). ADA may directly activate neutrophils and basophils (expressing FcγRIIA and FcγRIIIB receptors) and monocyte/macrophages (expressing FcγRIIA and FcγRIIIA receptors), leading to their release of PAF. In addition, FcγRIIA-expressing platelets may be involved by ADA-drug immunocomplexes, leading to their release of pathogenic serotonin (19).

## Prevention of HSR to Biologicals

### Identification of Patients With Clinical Risk Factors for HSR

The identification of those patients with higher risk for ADA development and HSR onset represents an important clinical issue. Risk assessment for biologicals-related HSR requires accurate evaluation of risk factors associated with immunogenicity.

Female patients seem to show a higher rate of biological-induced HSR, although this point remains a matter of debate (20). Atopic status, as well as previous adverse drug reactions, do not appear to be a clinical risk factor. In a multicenter cohort prospective study including 560 patients with different immune-mediated diseases and treated with eight different biologicals, immune-suppressants and antibiotics were associated with a decreased risk of ADA development, whereas smoking and infections during the study were associated with increased risk. Additionally, HLA-DQA1*05 was associated with a significantly increased rate of immunogenicity, although evidence to support genotyping strategy are lacking (21). The underlying disease itself, and in particular the highly activated B-lymphocyte status and the high expression of costimulatory molecules on dendritic cells in patients with immune-mediated diseases, can be an important factor in the development of an unwanted immune response towards the biological, as shown by the higher incidence of infliximab reactions in patients with rheumatoid arthritis than in those suffering from seronegative spondiloarthritis and vasculitis (22), or by the higher detection rate of anti-rituximab antibodies in autoimmune than in lymphoma patients (23–25).

Finally, taking a thorough history, including the course of treatment, represents the most useful risk-assessment tools in HSR to biologicals related to immunogenicity. In fact, intermittent therapy or re-exposure after a long treatment-free interval may be associated with an enhanced immune response (or loss of tolerance) to the biological agent, and thus, re-treated patients must be considered at risk of reactions (26–30).

### Assessment of Immunogenicity to Prevent Hypersensitivity Reactions to Biologicals

Using current technology and reagents, it is possible to develop highly sensitive ADA screening assays capable of detecting the most prevalent classes of ADA (IgM, IgG, and IgA); however, a separate IgE ADA assay may be required for IgE detection, due to the low circulating levels of specific IgE. It has been shown that ADA may be present in serum before therapy as in the case of cetuximab (pre-existing ADA), or developed during the course of treatment (induced ADA). Anyway, also in the case of induced antibodies, the development of ADA precedes the onset of reactions. In a longitudinal study performed in 91 infliximab-treated patients, assayed for ADA and drug levels by enzyme-linked immunosorbent assay and for IgE by ImmunoCAP system, it has been shown that the HSR tends to be preceded by ADA development, which in turn is associated with the reduction in drug serum levels (15). Specifically, all HSRs that occur after a period of drug interruption are preceded by ADA development.

Data from literature clearly show that the detection of pre-existing ADA IgE, in the serum of patients at baseline, is helpful to identify patients at risk of (severe) cetuximab-induced HSR (13). Finally, analyses stratified by ADA titer may identify patient subpopulations more at risk for clinical events (1). Overall, both ADA evaluation and therapeutic drug monitoring may have a relevant impact on clinical practice to prevent HSR to biologicals.

### The Role of Immunomodulatory Therapies to Block ADA Formation

Compared to biologic monotherapy, concomitant use of immunomodulators often increases the systemic exposure of the biologicals and decreases the formation of anti-drug antibodies, consequently enhancing clinical efficacy (31).

The use of methotrexate (MTX) may attenuate the frequency of ADA in patients suffering from rheumatoid arthritis, spondiloarthritis, and inflammatory bowel diseases. Azathioprine, also an immunosuppressive drug with a similar effect to MTX, has been observed with azathioprine usage in the management of Crohn’s disease, where it can be given in combination with infliximab or adalimumab to improve treatment and reduce immunogenicity and ADA formation. Some studies looked at concomitant therapy with leflunomide and mycophenolate that have also been shown to be associated with lower ADA prevalence, suggesting that all DMARDs may be associated with benefits against drug-induced immunogenicity (32). However, the definition of the impact of individual DMARDs on immunogenicity is still an unmet need in inflammatory arthritis because of small numbers of patients on DMARDs other than MTX, and because some patients were treated with more than one conventional DMARD. Of note, there have been studies indicating that addition of immunomodulators to the TNF-α inhibitors not only prevents the immunogenicity but also helps in the elimination of existing ADA, thus improving treatment and its safety (33–36). More research should be undertaken to identify and validate prognostic markers for predicting patients who would benefit the most and those who are at greater risk from combination therapy with immunomodulators and biologicals.

### The Role of Premedication

Antihistamines, corticosteroids, and acetaminophen are commonly used in premedication protocols to prevent HSR towards biologicals, with a high variability of protocols among centers (37). Among antihistamines, diphenhydramine and cetirizine are the most frequently administered, the latter favored by a lower degree of induced sedation. Most of clinicians use hydrocortisone before biological infusion, but 6-metyl-prednisolone is also administered. A placebo, randomized, controlled trial has previously shown that 200 mg hydrocortisone intravenously administered is able to reduce ADA development in a significant manner, although without totally abrogating HSR (38). Several studies have demonstrated a reduction in the number of HSR with the use of premedication, especially in cancer patients (39). However, in different clinical setting, the use of premedication is still controversial; despite some initial favorable results (40), most recent data obtained in both adult and pediatric patients suggest that premedication would not change the incidence of infliximab-related HSR (41–44). To date, there are no definitive data because a specific schedule has never been validated in controlled studies.

## Long-Term Management of Patients With HSR to Biologicals

Hypersensitivity reactions to biologicals may be characterized by severe anaphylaxis that can be rapidly progressing and fatal, and therefore establishing its cause is pivotal to long-term risk management, but more importantly, the optimal management of reactive patients must ensure the patient the most effective therapy for the treatment of the underlying disease in a safe manner.

### Definition of the Pathogenic Mechanism of HSR

Both skin testing and detection of serum ADA may be useful to define the pathogenic mechanisms in HSR patients. Skin testing represents the gold standard for the identification of true allergy (IgE-mediated) towards biologicals and are safe procedures also in patients who had experienced severe reactions (10). However, some main limitations and unmet needs are recognized: the low availability of test solutions, the lack of standardization of drug concentrations, and the unknown negativization rate (45).

Although they are not widely used, several commercial tests are available for the assay of ADA (CE marked), able to detect non-isotype-specific ADA. On the other hand, commercially available tests for IgE ADA detection are lacking, thus representing a crucial unmet need in the diagnostic workup of immediate HSR to biologicals (45). Other challenges in the IgE assay are represented by the low concentration of IgE compared with IgG antibodies, also with the same specificity, which may interfere with the IgE ADA assessment (46). Basophil activation test (BAT) could be included in the diagnostic workup of immediate HSR to show the ADA-mediated mechanism (both IgG and IgE) (47). However, studies in larger series of patients are needed to confirm the findings and establish BAT as a diagnostic tool, taking into account some technical limitations of BAT, mainly related to the existence of patients with non-responsive cells.

### Drug Desensitization to Induce Tolerance in Patients With HSR

Re-treatment with the same biological represents an option following an HSR, and drug desensitization (DD) is a therapeutic approach to safely administer biologicals causing an HSR. It is able to provide a temporary immune tolerance to drugs, and it is highly recommended when switching to alternate products with equal efficacy is not possible. Drug desensitization is a method largely applied for chemotherapy (48) and more recently used also for a safe reintroduction of biologicals in reactive patients; DD allows the administration of the full therapeutic dose in relatively short time (4–12 h), during which the biological is administered at increasing concentrations and increasing rate of infusion. Although different protocols have been published until now, the most frequently applied is the 12-step protocol (49). However, additional steps may be added, as well as other modifications may be performed regarding the time intervals between doses and the final rate of infusion. DD procedure has been demonstrated as a safe option for patients who have experienced HSR to cetuximab, rituximab, trastuzumab, anti-TNF blockers, and tocilizumab (50, 51). DD may be complicated by breakthrough reactions (45) that occur during the last steps and are usually mild and less severe than initial HSR (3). A correlation between breakthrough reactions and positivity of skin tests has been reported; in fact it has been described that a positive skin test result is the main predictor for breakthrough reactions (49).

Although DD is conceptually dedicated to patients in which an IgE-mediated mechanism is demonstrated by positive skin testing or serum IgE for culprit drug, patients with immediate HSR to chemotherapy (taxanes and platins) in which the IgE mechanism cannot be demonstrated have also been successfully desensitized (52).

There is as yet no consensus in literature about the underlying mechanisms operating in DD; the majority of data focused on the role of mast cells showing that desensitization procedure is associated with the inhibition of mast cell degranulation and cytokine production (53). Patients with IgE-dependent HSR displayed negative skin testing after DD, suggesting inhibition of the mechanisms that induce cell activation. These data have been extensively described in DD for chemotherapeutics and more recently for biological agents (54, 55) and show that DD is an antigen-specific process. Recent studies have shown that antigen/IgE/FcεRI surface expression do not change during the DD procedure and that mast cells’ hyporesponsiveness is attributable, at least partially, to abrogation of Ca++ mobilization, a critical determinant of both degranulation and cytokine production responses in mast cells (53). The adaptive immune response sustained by drug-specific T cells and its modification during DD procedures have been scarcely evaluated until now. Results obtained in patients submitted to DD for biologicals highly suggest that DD procedure may be able to modulate the adaptive immune response, with a decrease of ADA, including IgE isotype (53). More importantly, the modulation of humoral immune response is accompanied by the reduction of drug-specific T-cell proliferation to the biological (53, 54). Furthermore, the involvement of regulatory mechanisms, such as activation/expansion of drug-specific IL-35-producing T cells, may occur during the procedure and participate in the modulation of effector drug-specific response (56, 57).

## Conclusions

Biologicals are structurally immunogenic and are able to elicit a complete adaptive immune response, which negatively impacts their safety and may limit their clinical use. Specific diagnostic workup and a modified method of drug delivery (drug desensitization) are available in the clinical setting to manage patients with HSR. The knowledge of the mechanisms underlying HSR and biologicals’ immunogenicity is useful to increase the safety profile of current and novel biologicals.

## Author Contributions

AV and AM wrote the manuscript. EM, MP, FN, and EV revised the manuscript. All authors contributed to the article and approved the submitted version.

## Conflict of Interest

The authors declare that the research was conducted in the absence of any commercial or financial relationships that could be construed as a potential conflict of interest.

## Publisher’s Note

All claims expressed in this article are solely those of the authors and do not necessarily represent those of their affiliated organizations, or those of the publisher, the editors and the reviewers. Any product that may be evaluated in this article, or claim that may be made by its manufacturer, is not guaranteed or endorsed by the publisher.
